# Thinking About Suicide for a Long Time: A Scoping Review of Empirical Studies on Persistent Suicidal Ideation

**DOI:** 10.1111/sltb.70070

**Published:** 2025-12-16

**Authors:** Lena Spangenberg, Tobias Teismann, Inken Höller

**Affiliations:** ^1^ Department of Medical Psychology and Medical Sociology University Leipzig Leipzig Germany; ^2^ Mental Health Research and Treatment Center Ruhr‐University Bochum Bochum Germany; ^3^ DZPG (German Center for Mental Health), Partner Site Bochum/Marburg Bochum Germany; ^4^ Department of Clinical Psychology and Psychotherapy Charlotte Fresenius Hochschule Düsseldorf Germany

**Keywords:** chronic suicidal ideation, chronic suicide‐related thoughts and behaviors, persistent suicide‐related thoughts and behaviors, scoping review, suicidal ideation

## Abstract

**Introduction:**

Persistent suicidal ideation (PSI) represents a clinically relevant phenomenon that remains insufficiently understood. The aim of this scoping review was to provide an overview of empirical studies investigating PSI, with a focus on definitions, operationalizations, and associations with indicators of mental health, suicidal behaviors, and treatment approaches.

**Materials and Methods:**

Following established guidelines for conducting scoping reviews, a systematic search of the literature was conducted, and studies were screened according to predefined inclusion criteria.

**Results:**

*N* = 44 articles reporting on *n* = 40 individual studies were included in this review. The results indicate that several empirical studies have addressed PSI, most commonly using longitudinal designs. However, the absence of a consistent conceptualization across studies led to diverse and heterogeneous operationalizations and partly contradictory findings, making synthesis difficult.

**Discussion:**

A major limitation of this review lies in the inconsistent terminology across publications, which may have resulted in missed studies. Furthermore, case studies and theoretical works were excluded, narrowing the scope of the findings.

**Conclusion:**

The clinical relevance of PSI contrasts with the lack of empirical findings addressing its epidemiological and phenomenological characteristics. Future research should establish a common definition and operational criteria for PSI.

## 1 Introduction

Suicidal ideation is a frequent and burdensome experience. In a German representative study, 8% of respondents reported such thoughts within the past 2 weeks (Forkmann et al. 2012), and prevalence among people with mental illness has been estimated between 16% and 67.5% (Teismann et al. 2024). Beyond being a key risk factor for suicidal behavior (Franklin et al. 2017), suicidal ideation itself can weigh heavily on those affected (Jobes and Joiner 2019). Evidence shows that while suicidal ideation often fluctuates over hours and days (Hallensleben et al. 2018), for many it also persists over months or years. Inpatients with treatment‐resistant disorders, for instance, required a median of almost 9 years for remission, and fewer than half achieved remission from suicidal ideation at all (Perry et al. 2009). This persistence and recurrence highlight suicidal ideation as a major clinical challenge, especially because it often only inadequately responds to treatment (DeCou et al. 2019).

To our knowledge, no generally accepted definition of chronic, persistent, or recurrent suicidal ideation exists. There is no consensus on the criteria or operationalization of the phenomenon, but it has been noted that the term chronic may be stigmatizing (Teismann et al. 2025). In the following, we will consequently refer to persistent suicidal ideation (PSI), even if other terms have been used to describe the phenomenon of interest.

Studies on the association between PSI and the prevalence of suicidal behavior are still rare; the same applies to studies on risk factors for a persistent course of suicidal ideation and investigations into the mechanisms maintaining PSI (Hennings 2022; Hensel and Teismann 2026). Nevertheless, it is known from clinical practice that PSI is often present in the context of borderline personality disorder, persistent depression, chronic pain disorders, and persistent trauma disorders (see Kirtley et al. 2020; Paris 2019). A study by Höller et al. (2025a) highlights that PSI is a frequent and clinically relevant issue in outpatient care. Most mental health professionals (86.5%) reported having worked with patients with PSI and estimated its 12‐month prevalence at 14.4%.

### 1.1 Objectives

Although there is compelling research (as well as clinical experience) that points to the relevance of PSI, the current state of research on this issue appears limited. A detailed summary of key findings from available studies on PSI would be an important starting point for further research on this topic, with a view to compiling systematic recommendations for considering PSI in clinical risk assessments and treatment planning. Against this background, we conducted a scoping review to explore the extent, range, and nature of relevant research activity and to summarize research findings on PSI. Within the scoping review, we were interested in the following aspects concerning PSI: (1) definitions, conceptualization, and assessment, (2) phenomenology and prevalence, (3) associations (i.e., antecedents, correlates, and consequences) with indicators of mental health (e.g., risk as well as protective factors such as psychopathological symptoms or social support), (4) associations with previous, current, or future suicide‐related thoughts and behaviors (STBs), and (5) impact on treatment and intervention strategies.

## 2 Materials and Methods

This scoping review followed established best practice guidelines for systematic scoping reviews (Arksey and O'Malley 2005; Peters et al. 2022). The search process was conducted in line with the PRISMA‐ScR framework (Tricco et al. 2018). The study's aim and planned methods were preregistered on the Open Science Framework prior to initiating the search (Höller et al. 2025b).

### 2.1 Search Strategy

We searched for articles on persistent suicidal ideation that were published until March 1st 2025, using the electronic library databases PubMed, Psychinfo, Web of Science, and Google Scholar. The search strategy included the following terms: (1) “chronic suicid*” OR “persistent suicid*” OR “constant suicid*” OR “recurrent suicid*,” (2) “treatment refractory suicid*,” and (3) “treatment resistent suicid*.” Notably, the last two terms did not yield any results in any of the databases mentioned above. The Boolean operator OR was used to combine the search terms as outlined above. In Web of Science, the search was performed using the Topic Search (TS =) for the first term (1), which includes titles, abstracts, and keywords. No controlled vocabulary was applied, and the search was conducted across all database fields without limiting the search to titles or abstracts, except for Web of Science, as mentioned above. Furthermore, we screened reference lists of included articles, and articles that met the inclusion criteria were added to the review evidence base.

### 2.2 Inclusion and Exclusion Criteria

Articles and publications were initially deemed to be eligible if they fulfilled the following inclusion criteria: (1) quantitative and qualitative research studies, opinion papers, commentaries, editorials, and letters, (2) published in or translated to English, (3) published in a peer‐reviewed journal, (4) all ages, all nationalities, all genders, all sample types (e.g., clinical, general population, online), and (5) an explicit focus on PSI (i.e., mention of the search terms in keywords, title, or abstract). Publications were excluded in the following cases: (1) non‐peer‐reviewed articles, (2) articles not published in English, (3) poster abstracts, gray literature, and unpublished work, conference proceedings, conference abstracts, and (4) studies that did not explicitly focus on PSI.

Reflecting the iterative nature of scoping reviews, amendments of these criteria were possible throughout the study selection phase.

### 2.3 Study Selection

**FIGURE 1:**
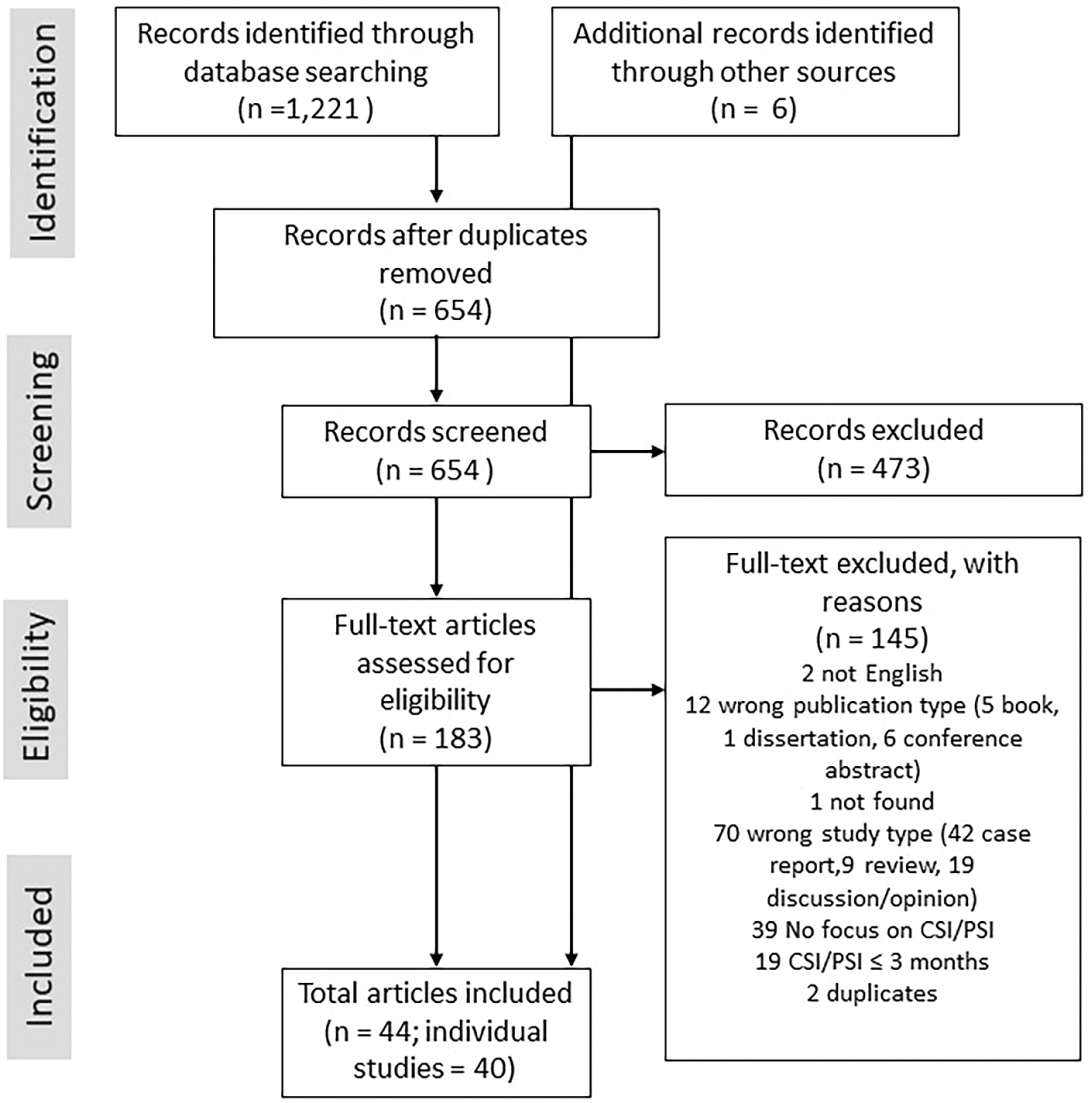
Flow chart of the selection process.

First, a study assistant removed duplicates. Second, using the inclusion and exclusion criteria described above, LS, IH, and TT independently screened titles and abstracts of all retrieved articles using the internet tool Rayyan (https://www.rayyan.ai). Third, the full texts of the remaining studies were assessed against the outlined criteria. During this step, the inclusion and exclusion criteria were refined. In light of the large heterogeneity, we decided to exclude (1) case reports, (2) nonempirical papers (i.e., theoretical papers, opinion papers, editorials, and reviews), (3) studies lacking any definition or operationalization of PSI, and (4) studies that used a time criterion of < 3 months to define PSI. This decision was based on the time criteria used in the DSM‐5 to define chronic conditions (which are not < 3 months). In case of uncertainty about study eligibility, LS, IH, and TT discussed the study against the eligibility criteria for inclusion or exclusion and reached a consensus. During this process, it was decided to include two qualitative studies that offered valuable insights in clinicians' and patients' views on the topic despite the lack of a definition or operationalization of PSI. See Figure 1 for the flow chart of the selection process.

### 2.4 Charting the Data

A Microsoft Excel data charting template was used. Data was extracted independently by LS, IH, and TT and included the following: author(s), year, country, title, article type, study aim/hypotheses, study population and sample size, study design, definition and conceptualization of PSI, measure to assess PSI, indicators of mental health, measures to assess suicide plan/suicide attempt/suicide, key findings, and study limitations. Critical assessment of the quality of the included studies is not a scoping review requirement (Arksey and O'Malley 2005; Peters et al. 2022) and was not part of the process.

The draft extraction tool was piloted on a subset of sources to be included in the review to test its feasibility for the review.

### 2.5 Results Synthesis

LS, IH, and TT conducted the synthetization of results jointly using a step‐wise approach: The first step was the categorization of articles based on predefined themes. As such, findings were grouped under the aforementioned headings: (1) definitions and conceptualization, (2) phenomenology and prevalence, (3) associations with indicators of mental health, (4) associations with suicide plans, attempts, and suicide (i.e., suicidal behavior), and (5) impact on treatment and intervention strategies. Results of this step of the synthesis were presented in either tables or text. The authors of the scoping review discussed how well the sub‐groupings fit the data.

## 3 Results

Of 1221 records that were initially identified, 183 were accessed for full‐text screening after removal of duplicates and initial screening. Following full‐text screening, 38 articles remained that are included in the review. An additional six articles were identified through screening of the reference lists of these articles and were also included, leading to a total number of 44 articles included in the review. See Figure 1 for the flow chart of the selection process.

### 3.1 Description of Studies

The 44 articles reported on 40 individual studies (i.e., five articles stemmed from the same trial and therefore the study is counted as one). Table 1 presents the included articles and their methodological characteristics. Of those, 52.5% (*n* = 21) were conducted in non‐clinical populations (*n* = 11 community/general population, *n* = 1 soldiers, *n* = 8 adolescents/students, *n* = 1 primary care staff) and 47.5% (*n* = 19) in clinical populations (*n* = 8 affective disorders, *n* = 1 PTSD, *n* = 1 HIV, *n* = 3 psychotic episode, *n* = 1 OCD, *n* = 4 transdiagnostic psychiatric sample, *n* = 1 traumatic brain injury). Most studies (40.0%) were carried out in either the USA (*n* = 14), Canada (*n* = 2), or European countries (35.0%, *n* = 14), whereas fewer were from Asian countries (17.5%, *n* = 7) or Australia (5.0%, *n* = 2).

**TABLE 1:** Study characteristics and operationalization and prevalence of PSI.

Authors	Year	Country	Population	Sample size (age, gender)	Operationalization of PSI (term used in the article)	Time criterion (no. of assessments)	Prevalence of PSI
Cross‐sectional studies							
Sasaki et al.	2022	Japan	Non‐clinical, employees	*n* = 12.248 (20–65 years, 57.9% male)	SI present for “long time” (persistent SI)	Vague (1)	8.5%
Cohen et al.	2020	USA	Non‐clinical, community sample (minor attracted persons)	*n* = 333 (mean age 36.7 years, 94.8% male)	SI present for many years (chronic SI)	Vague (1)	38.1%
Garakani et al.	2022	USA	Clinical, patients with mood disorder	*n* = 86 (18–69 years, 39% male)	See text (chronic SI)	24 months (1)	
Nock et al.	2018	USA	Non‐clincal, soldiers	*n* = 3.501 (no information on age, gender)	SI beyond year of onset, annual proportional PSI = percentage of years with SI since year‐of‐onset (persistent SI)	12 months (1)	52.2%, proportional PSI median = 33% (i.e., for many years)
Voss et al.	2019	Germany	Non‐clinical, adolescents/young adults	*n* = 1.180 aged 14–21 (mean age 17.9, 51.7% male)	SI with duration > 1 year (time in years between onset and last occurrence) (persistent SI, recurrent SI)	12 months (1)	7.5%
Sivak et al.	1999	USA	Clinical, veterans with PTSD	*n* = 100 (100% male)	See text (chronic SI)	Not given (1)	13.4% daily SI
Longitudinal studies							
Buddeberg et al.	1996	Switzerland	Non‐clinical, community sample (students)	*n* = 475 (mean age 16.0 years, 37.6% male)	SI at three assessments (BL and FU at 12 and 18‐months) (persistent SI)	18 months (3)	9.8%, 12.4% in females, 5.6% in males
Clark et al.	2022	USA, Sweden	Non‐clinical, general population	*n* = 1.610 (17–34 years; 5.6% gay/lesbian, 13.5% plurisexual, 80.9% heterosexual; 50.3% cisgender woman, 48.1% cisgender men, 1.6% transgender/nonbinary)	SI at two assessments (BL and FU at 12 months) (persistent suicidality, chronic)	12 months (2)	35% in sexual minorities, 15.0% in heterosexuals
Guo et al.	2023	China	Non‐clinical, students	*n* = 4.225 (10–19 years, 50.7% male)	SI at two assessments (BL and FU at 12 months) (persistent SI)	12 months (2)	1.8%, 1.4% in males, 3.4% in females
Beaudequin et al., Can et al., Dutton et al.^a^	2021–2023	Australia	Clinical, patients with MDD	*n* = 32 (mean age 45.7 years, 53.1% female) with MDD; n 0 29 with EEG data in Can et al. 2023	Clinical evaluation (chronic suicidality, persistent SI)		
Seo et al.	2014	Korea	Clinical, patients with depression and SI	*n* = 565 (mean age 44.7 years, 27.0% male)	SI at several assessments at (week 0, 6, 12) (persistent suicidality/SI)	12 weeks (3)	77.2%
Willkens et al.	2024	USA, UK, Tanzania	Clinical, patients with HIV/non‐clinical, community controls	*n* = 884 (423 with HIV, 461 community controls; median age 35 years, 69.3% male)	SI at three assessments (BL and FU at 12 and 24 months) (persistent SI)	24 months (3)	2.4% (CC) 5.7% (HIV), 3.3% male, 4.2% female
Kivelä et al.	2019	NL	Clinical, patients with SI	*n* = 230 (mean age 41.4, 33% male)	SI at two consecutive points (year 6, 9) (persistent SI, chronic SI)	36 months (2)	3.0%
Wilcox et al.	2010	USA	Non‐clincal, students	*n* = 1.253 (17–19 years in year 1, 47.3% male)	SI at two assessments (baseline, FU in year 1, 2, 3, or 4) (persistent SI)	48 months (5)	2.6%
Sicotte et al	2023	Canada	Clinical, patients with first psychotic episode	*n* = 382 (mean age 23.5 years, 78.0% male)	SI over 5 years (annual assessment) (persistent SI)	60 months (5)	7.1%
Alonso et al.	2010	Spain	Clinical, patients with OCD	*n* = 218 (mean age 31.3 years, 57.3% male)	SI at three consecutive assessments (FU every 3 months for 1–4 years) (persistent SI)	9 months (3)	8.2%
Klimes‐Dougan et al.	2008	USA	Non‐clinical, offspring of depressed and well mothers	*n* = 139 adults (mean age 22 years, 42% male)	Proportion of assessment waves with suicidal content (persistent suicidality)		
Vasiliadis et al	2013	Canada	Non‐clincal, community sample	*n* = 2.494 (> 65 years; 42.0% male, 80.3% with follow‐up data after 1 year)	SI at two assessments (BL and FU) (persistent suicidal thoughts/SI)	12 months (2)	1.3% of follow‐up sample (20.3% of those with baseline SI)
Nobile et al.	2021	France	Clinical, BD I, II or NOS with current depression	*n* = 938 (45.6% with 1 year follow‐up, 28.6% with 2 year follow‐up; mean age 40.7 years, 37.6% male)	SI at three assessments (BL and FU in year 1 and 2) (persistent SI)	24 months (3)	
Hintikka et al	2001	Finland	Non‐clinical, general population	*n* = 1.593 (mean age 45.9 years for males [41.9%], 44.5 years of females)	SI at two assessments (BL and FU) (persistent SI)	12 months (2)	7.3% (10.1% in males, 5.4% in females)
Wolff et al.	2018	USA	Clinical, psychiatric inpatients	*n* = 104 (mean age 14.9 years, 27% male; 77% with complete data on all three assessments)	SI at three assessments (BL and FU at 3 and 6 months) (chronic SI, persistently suicidal, persistent SI, chronically elevated SI)	6 months (3)	23.1% chronic SI trajectory
Pan et al.	2017	Korea	Non‐clinical, adolescents	*n* = 1.710 (mean age 13.3 years, 49.6% male)	SI at three assessments (BL and FU at year 1 and 2) (persistent SI)	24 months (3)	
Brezo et al.	2007	Canada	Non‐clinical, adolescents	*n* = 2.000, some followed up in wave 2 and 3	SI at BL and one FU (in wave 1 or 2) (persistent SI, chronic SI)	Unclear (3)	13.0% (in lifetime ideators)
Zhang et al.	2011	Hong Kong	Non‐clinical, general population	*n* = 997 (aged 20–59)	Lifetime SI (BL) and SI at FU (persistent suicidality, SI)	12 months (2)	6.2%
Gambadauro et al.	2020	6 european countries	Non‐clinical, adolescents	*n* = 1.958 (mean age 15.35, 43.2% male, 10.9% sexual minority youth)	SI at ≥ 2 assessments (BL and FU at 2 and 4 months) (persistent suicidality, SI)	4 months (3)	
Kim et al.	2009	Korea	Non‐clinical, students	*n* = 1.666 (mean age 13.1 and 14.1 years, 55.2% male)	SI at two assessments (BL and FU) (persistent suicidality, persistent SI)	8–10 months (2)	
Borges et al.^b^	2008	USA	Non‐clinical, general population	*n* = 5.877 (15–53 years), 87.6% with FU	Lifetime SI (BL) and SI during FU (persistent SI)	120 months (2)	35.0%
Perry et al.^b^	2009	USA	Clinical, patients with treatment refractory disorders	*n* = 226 (mean age 31.0 years, 75.2% female)	SI at BL and last FU (13 FUs. biannually for 5 years, annually for 3) (persistent SI)	Not given (2)	53.2 (baseline ideators), 35.2%
Rath et al.^b^	2021	Germany	Clinical, patients with STBs	*n* = 308 (mean age 35.9, 46.4% male)	SI at five assessments (BL and FU every 3 months) (persistent SI, chronic SI)	12 months (4)	12.6% (27% of those with complete FU data)
Teismann et al.^b^	2016	Germany	Non‐clinical, general population	*n* = 1.389 (mean age 20.7, 0% male)	SI at two assessments (BL and FU) (persistent SI)	17 months (2)	5.4%
ten Have et al.^b^	2009	NL	Non‐clinical, general population	*n* = 4.848 (18–64 years)	SI at two FUs (unchanged, unfavorable course of SI)	36 months (3)	31.3%
Trajectory modeling							
Choi et al.	2023	Korea	Non‐clinical, general population with SI	*n* = 10.017 (> 20 years)	SI at BL and one FU, Mixture modeling of trajectories (persistent SI)	96 months (8)	3 SI trajectories, 0.9% high‐persistent SI
Madsen et al.	2019	Denmark, USA	Clinical, patients with MDD	*n* = 811 (mean age 42.5 years, male 56.4%)	SI assessed at BL and 12 weekly FU, Growth Mixture Modeling of Trajectories (persistent SI)	12 weeks (13)	5 SI trajectories, 9.8% persistent‐high SI
Gohar et al.	2023	Norway, Denmark	Clincal, patients with first psychotic episode	*n* = 301 (mean age 27.8 years, 58% male)	SI assessed at BL and 2 FU at year 1 and 2, Growth Mixture Modeling of Trajectories (chronic SI, persistent SI)	24 months (3)	4 SI trajectories, 15% stable SI, 6% worsening SI 6%
Madsen et al.	2016	Denmark	Clinical, patients with first psychotic episode	*n* = 521 (18–45 years)	SI assessed at BL and FU at year 1 and 2, Growth mixture modeling of trajectories; PSI = SI at all assessments (chronic SI, chronically elevated SI, persistent SI)	24 months (3)	PSI 40% during the first 2 years, 3 SI trajectories (PSI more likely in those with frequent‐stable SI (33%) or frequent‐increasing SI (6%))
Köhler‐Forsberg et al.	2017	Denmark, USA	Clinical, patients with BD I or II	*n* = 482 (mean age 38.9 years, 41.3% male)	SI over 9 assessments (BL and FU) (persistent SI)	6 months (9)	14.0%; 4 SI trajectories, 11.1% moderate‐stable, 2.9% moderate‐unstable
Kasckow et al.	2016	USA	Clinical, patients with MDD	*n* = 468 (mean age 69.3 years, 35.05% male)	SI at several assessments (week 0, 1, 2, 4, 6, 8, 10, and 12–16), Latent Class Growth modeling of trajectories (persistent SI)	16 weeks (8)	4 SI trajectories, 6.4% high and persistent SI, 16.5% low SI
Czyz and King^b^	2015	USA	Clinical, adolescents with STBs	*n* = 376 (13–17 years, 28% male)	SI assessed at BL and FU at months 3, 6, and 12, Latent Class growth modeling (persistent SI, chronic SI)	12 months (4)	3 SI trajectories, 10.9% PSI group
Qualitative studies							
Knight et al.	2020	Australia	Clinical, patients with traumatic brain injury	*n* = 19	Unclear (chronic SI)		
Dobscha et al.	2023	USA	Non‐clinical, primary care/mental health staff	*n* = 40	Unclear (chronic SI)		

Abbreviations: BL, baseline; FU, follow‐up; SI, suicidal ideation.

^a^ Articles based on the same trial and study sample.

^b^ Additionally identified.

The majority of studies (80.0%, *n* = 32) had a longitudinal design; few were cross‐sectional (15.0%, *n* = 6), and two had a qualitative approach (5.0%, *n* = 2).

### 3.2 Definition and Operationalization

Of the included articles, 7.5% (*n* = 3, Beaudequin et al. 2020; Garakani et al. 2022; Sivak et al. 1999) defined PSI or provided a conceptual description. For example, Beaudequin et al. (2020) described PSI as “experiencing SI on a continuous or intermittent basis over a period of time (months, years) without the immediate risk implied by acute suicidality.” In contrast, Sivak et al. (1999) referred to PSI as “ongoing, transient, intermittent thoughts of ending one's life” (cf. Motto 1992). Another attempt at a definition has recently been proposed by Garakani et al. (2022). The authors define chronic suicidal ideation as “the presence of suicidal ideation lasting 24 months or longer, occurring either within or outside of a depressive episode, consisting of cognitive distortions, conscious, compulsive, and rumination‐type death thoughts, but not reaching the level of active suicidal ideation, intent, or plan” (p. 162).

The majority of studies (62.5%, *n* = 25) used the term “persistent,” while fewer employed “chronic” (12.5%, *n* = 5). 22.5% of studies (*n* = 9) used ≥ 2 terms somewhat interchangeably (i.e., chronic, persistent, recurrent, chronically elevated), and one referred to an unchanged/unfavorable course of SI (see Table 1). The majority of the reviewed studies provided an *operationalized* definition of PSI (70.0%, *n* = 28), which usually specified that suicidal ideation had to be present at multiple assessments during the study or for a specific period of time (if assessed retrospectively). Few studies relied on a single, retrospective assessment of suicidal ideation (15.0%, *n* = 6; with *n* = 3 studies specifying no or a vague time criterion such as “a long time” or “for many years”; see Table 1). The majority of studies also conducted multiple assessments of suicidal ideation (57.5%, *n* = 23), with the number of assessments ranging from two to 13. Regarding the time criterion, 15.0% (*n* = 6) stated that suicidal ideation had to be present for < 12 months, whereas roughly half of the studies (47.5%, *n* = 19) specified a time criterion of ≥ 12 months. Of all 40 included articles, 20.0% (*n* = 8) provided either a vague (*n* = 2) or no (*n* = 6) time criterion for the duration that suicidal ideation had to be present in order to be persistent. See Table 1 for details. In Table S1, all instruments and items used by the studies to assess STBs are displayed.

### 3.3 Prevalence and Phenomenology

Of all studies, 62.5% (*n* = 25) reported prevalence rates for PSI (or numbers that enable the calculation of prevalence rates). Depending on the sample, the operationalization of PSI, and other factors (e.g., how many people participated in the follow‐ups), these prevalence rates vary considerably and are difficult to compare between studies. For example, in a community sample, the rate for PSI was 1.3% (with two assessments over 12 months; Vasiliadis et al. 2013), while in patients with major depression and suicidal ideation, the rate for PSI was 77.2% (three assessments over 12 weeks; Seo et al. 2014). See Table 1 for a detailed overview.

Regarding the phenomenology of PSI, Knight et al. (2020) reported from qualitative interviews with participants that suicidal ideation is “always there,” but there are “peaks and throughs in the context of triggers.” Seven studies (17.5%) examined trajectories of suicidal ideation over multiple assessments, finding between three and five distinct trajectories over time periods of 16 weeks up to 5 years (Köhler‐Forsberg et al. 2017; Madsen et al. 2016; Choi et al. 2023; Kasckow et al. 2016; Gohar et al. 2023; Czyz and King 2015; using subtyping approaches such as growth mixture modeling or latent class analyses). In most studies, there were one to three trajectories characterized by suicidal ideation that was either persistently high (e.g., Choi et al. 2023; Czyz and King 2015) or stable at a low or moderate level (e.g., Madsen et al. 2016; Gohar et al. 2023).

### 3.4 Associations With Indicators of Mental Health

#### 3.4.1 Antecedents of PSI

The reviewed studies presented a number of factors as predictors of PSI (group membership/proportion) in follow‐ups of varying length. These include clinically diagnosed psychiatric disorder/history of psychiatric disease (Guo et al. 2023; Sasaki et al. 2022), depressive symptoms (Guo et al. 2023; Wilcox et al. 2010; Köhler‐Forsberg et al. 2017; Gohar et al. 2023), maternal history of depression (Wilcox et al. 2010), bipolar disorder (Nock et al. 2018), panic disorder (Nock et al. 2018), current PTSD diagnosis (Köhler‐Forsberg et al. 2017), anxiety (Seo et al. 2014), ADHD (Nock et al. 2018), and a longer duration of untreated psychosis (Gohar et al. 2023). Further factors were substance abuse (Nock et al. 2018; Sicotte et al. 2023; Gohar et al. 2023), sleep problems (Guo et al. 2023; Kivelä et al. 2019), low social support (Guo et al. 2023; Wilcox et al. 2010), attachment problems (Guo et al. 2023), childhood sexual abuse/exposure to domestic violence (Wilcox et al. 2010; Klimes‐Dougan et al. 2008), premorbid childhood social adjustment (Gohar et al. 2023), hopelessness (Kivelä et al. 2019), use of other antidepressants than SSRI, nonacceptance of emotional responses, and limited access to emotion regulation strategies (Wolff et al. 2018), and previous suicide attempts (Köhler‐Forsberg et al. 2017) as well as suicidal ideation (Seo et al. 2014).

#### 3.4.2 Correlates of PSI

Additionally, PSI was associated with current or lifetime affective disorder (Alonso et al. 2010), general mood disorder and major depression (Sivak et al. 1999; Seo et al. 2014), recurrent depression (Seo et al. 2014), current chronic PTSD (Sivak et al. 1999), anxiety (Zhang et al. 2011), Avoidant Personality Disorder (Alonso et al. 2010), childhood sexual abuse/exposure to domestic violence (Cohen et al. 2020), internalizing or externalizing problems (Klimes‐Dougan et al. 2008), hopelessness and life distress (Zhang et al. 2011). Other correlates of PSI were alcohol use (Willkens et al. 2024), prior psychiatric hospitalization (Cohen et al. 2020), chronic pain, stressful life events, feelings of entrapment, poor self‐worth, hidden disability, social exclusion (Knight et al. 2020), and early age of onset of suicidal ideation as well as a history of suicide attempts (Seo et al. 2014).

#### 3.4.3 Consequences of PSI

Participants with PSI showed higher levels of depression (Buddeberg et al. 1996; Alonso et al. 2010; Klimes‐Dougan et al. 2008; Hintikka et al. 2001), anxiety, disturbed eating behavior, somatization, obsessive/compulsive behavior, and physical impairment (Buddeberg et al. 1996) than participants without PSI.

Compared to members of other suicidal ideation subgroups (minimal, low, or rapidly decreasing suicidal ideation), participants in the PSI group were more likely to be affected by cognitive impairment (e.g., lower inhibition, lower attention, lower global functioning) than those in the control group (Kasckow et al. 2016).

### 3.5 Protective Factors

Only few studies reported associations with variables that improved PSI or exerted a positive effect on it (*n* = 3). Knight et al. (2020) qualitatively investigated protective factors in traumatic brain injury patients. They found that social support, spirituality, and positive personal attributes (e.g., humor, resilience, acceptance, empathy) as well as hope were protective factors against PSI. Zhang et al. (2011) also highlighted hope as a protective factor in a non‐clinical sample. In another study that focused on veterans with PTSD, Sivak et al. (1999) found that counter‐suicidal cognitions protect against PSI (i.e., related to family/friends 76.3%, faith/spirituality 35%, work/financial responsibilities 10%).

### 3.6 Associations With STBs

A series of prospective studies conducted on various populations (patients diagnosed with major depression, OCD, or first‐episode psychosis, adolescents/young adults) consistently came to the conclusion that PSI is associated with a heightened risk of suicide attempts (Alonso et al. 2010; Brezo et al. 2007; Czyz and King 2015; Madsen et al. 2016, 2019; Sicotte et al. 2023; Wolff et al. 2018) and suicides (Wolff et al. 2018; Alonso et al. 2010; Gohar et al. 2023) during the study period. In a treatment study of patients suffering from OCD, for example, 72.2% of individuals who suffered from PSI attempted suicide or died by suicide during the study period of 1–6 years (Alonso et al. 2010). In a study of adolescents/young adults who were examined over a period of 6 months after discharge from inpatient treatment, 46% of those with PSI attempted suicide (Wolff et al. 2018). Furthermore, PSI was found to predict suicidal ideation 8 years later (Gohar et al. 2023). Finally, previous suicide attempts were identified as predictors of a persistent course of suicidal ideation (Madsen et al. 2019).

### 3.7 Treatment

A pilot study investigated the effectiveness of 6 weeks of oral ketamine administration in the treatment of patients suffering from PSI (Can et al. 2021, 2023; Dutton et al. 2022). It was found that ketamine administration led to a reduction in suicidal ideation at the posttreatment assessments after 6 weeks and the follow‐up assessment after 10 weeks. In addition, effects were found on depression, anxiety, and, above all, stress (Dutton et al. 2022). Further studies examined various treatment‐seeking populations (Alonso et al. 2010; Brezo et al. 2007; Czyz and King 2015; Madsen et al. 2019; Sicotte et al. 2023; Wolff et al. 2018), but none of the included studies investigated the extent to which *specific* interventions are effective in reducing PSI. In a qualitative study, clinicians who were asked about the use of structured suicide risk assessments stated that repeated questioning in the context of PSI offers no additional diagnostic benefit and takes up time that is then not available for therapy (Dobscha et al. 2023). However, given the association between PSI and suicide attempts, Sicotte et al. (2023) nonetheless note that repeated risk assessments should not be dispensed in cases of PSI.

## 4 Discussion

Our scoping review showed that PSI has been empirically investigated in a whole series of studies and is associated with a variety of mental health indicators, such as risk as well as protective factors, and STBs. At the same time, PSI has been named, defined, and assessed in very different ways, making it impossible to make generalizing statements at this point in time.

For example, the definition of Garakani et al. (2022; see above) falls short in including persons that transition to suicidal intentions, plans, or actions *while* having persistent suicidal thoughts. While individuals can continuously experience suicidal thoughts with only mild or stronger fluctuations around a homeostatic equilibrium point for longer periods, these thoughts can nonetheless dynamically and rapidly transition into suicidal action (Bryan et al. 2020). Most studies use the term “persistent” which aligns with the findings of a recent survey of German experts in suicide research and treatment. The experts favored the term “persistent” over chronic since it appears less stigmatizing and less likely to imply that the respective thoughts are unchangeable (Teismann et al. 2025). Based on this expert survey, the following working definition of persistent suicidal ideation was derived: “Persistent suicidal ideation refers to thoughts and ideas that a person has about ending their own life, which occur on most days (per month) over a period of 12 months or longer” (Teismann et al. 2025). However, further (empirical) confirmation of the validity of the definition is still pending.

Overall, this review underlines that available *empirical* evidence on PSI is not scarce but strongly limited by the lack of a uniform approach toward naming and conceptualizing PSI, accompanied by a correspondingly heterogeneous operationalization of PSI. Since the reviewed studies differed so greatly in their methodological approach to the topic (i.e., design, time criteria, assessments), it is difficult, if not impossible at this point, to draw general conclusions regarding the prevalence of PSI in various clinical and non‐clinical populations or the associations with indicators of mental health/STBs. Given the obvious clinical relevance of PSI in specific patient groups (e.g., borderline personality; Sansone 2004), and the prevalence of PSI in outpatient settings as estimated by clinicians (e.g., 14.4%; Höller et al. 2025b), these gaps in knowledge are striking.

While qualitative research pointed out that PSI is characterized by its presence for most, if not all the time (Knight et al. 2020), most studies cannot provide empirical evidence due to methodological limitations. In other words, it appears specifically difficult to make assumptions about the *continuous* presence or intensity of suicidal ideation over the examined time frames. Studies often rely on either single, retrospective assessments or repeated (retrospective) assessments of suicidal ideation at follow‐ups (that either reflect current suicidal thoughts or relate to a previous time span of weeks or years). Although the term persistent (or chronic) is used, the assessments might not be able to capture the “true” amount of suicidal ideation during longer time intervals, which seems likely in light of potential recall biases (Gratch et al. 2021; Trull and Ebner‐Priemer 2013) and differing disclosure of suicidal ideation (Hallford et al. 2023). To date, (P)SI is measured repeatedly at selected timepoints and it remains ultimately unstudied if suicidal ideation is indeed present most of the time (i.e., is truly persistent) or fluctuates (i.e., vanishes and reoccurs and is rather recurrent than persistent; Voss et al. 2019). There are only few studies with high temporal resolution (i.e., using ecological momentary assessments) that examine the course of suicidal ideation over longer time frames such as weeks or months (Kivelä et al. 2022). Future studies should use such methods because they are a requisite to understanding the persistence of suicidal ideation based on empirical data. The prevalences found by the reviewed studies may consequently tend to over‐ or underestimate the proportion of individuals experiencing PSI, which seems very likely given the considerable range of prevalence rates across studies. The interpretation of prevalence rates is further complicated by the fact that in longitudinal studies some cases are usually lost at follow‐up (eventually the most severe cases, e.g., Kivelä et al. 2019; Gambadauro et al. 2020) and missings are only occasionally imputed (e.g., Choi et al. 2023). Since the studies examining trajectories of suicidal ideation in various clinical groups have demonstrated that there is at least one profile characterized by the presence of suicidal ideation (on either a high, moderate, or low level), it can also be concluded that PSI is relevant for a certain proportion of patients.

Despite the incomplete knowledge regarding the prevalence and phenomenology of PSI, the reviewed studies clearly show that PSI is associated with numerous negative indicators of mental health. Additionally, psychiatric diagnoses have been reported to be associated with PSI, with depression being the most frequently named (e.g., Zhang et al. 2011; Nock et al. 2018). Furthermore, deficient emotion regulation (Wolff et al. 2018), physical impairment (Buddeberg et al. 1996), and cognitive impairment (Kasckow et al. 2016) are associated with PSI.

While many other associations with psychopathological or somatic symptoms have been identified, research related to protective factors is absolutely scarce. Yet, there is some evidence that factors such as social support, spirituality, positive personal attributes, and counter‐suicidal cognitions may protect against PSI (Knight et al. 2020; Sivak et al. 1999).

However, results are difficult to interpret due to some very small sample sizes, some very specific samples (e.g., traumatic brain injury patients, veterans), inconsistent or no definition of PSI, and different time and assessment frames. The findings underline that too little is known about associations with risk factors and even less is known about associations with protective factors or possible mechanisms involved in the development, maintenance, and eventually remission of PSI. It remains unclear whether suicidal ideation persists in the context of an ongoing mental disorder or becomes an independent psychopathological process (i.e., such as suicide‐related rumination; Hensel et al. 2024). Against this background, Paris (2007) describes the inner experience of those affected by PSI as characterized by three modes: psychological pain, inner emptiness, and hopelessness. People with PSI thus seem particularly burdened by internal and external stressors.

A series of longitudinal studies has consistently shown that PSI is associated with an increased risk of suicide attempts and suicides. This association has been observed in various patient populations (Alonso et al. 2010; Madsen et al. 2019; Sicotte et al. 2023) as well as in studies of adolescents (Brezo et al. 2007; Czyz and King 2015; Wolff et al. 2018). It can be assumed that there is an overlap between individuals who suffer from PSI and individuals who attempted suicide multiple times. Studies on people with multiple suicide attempts—who most likely represent a subset of those with PSI—found that these individuals suffer from more severe symptoms, higher rates of comorbidity, and substance abuse than lifetime single attempters (Forman et al. 2004; Rudd et al. 1996). However, there is currently a lack of studies that examine this association in more detail. Furthermore, it is unclear whether and to what extent recurrent versus PSI is associated with a different risk of suicidal behavior. As such, caution should be exercised in assuming a reduced risk of suicidal behavior in the context of PSI (cf. Garakani et al. 2022). Instead, it seems helpful to refer to an analogy by Joiner (2014) “For example, there are skin cells that are precancerous and yet, over decades, do not develop into cancer. It would be regrettable if a dermatologist, upon detecting a precancerous cell, said, ‘oh, let's not worry about it, if it were going to run into cancer it already would have’. It would also be contraindicated to immediately conduct highly invasive surgery. What does the competent dermatologist do instead? Relatively moderate measures like in‐office removal of the affected area, and, in a phrase, ‘watchful waiting’ – that is to say. Periodic monitoring of the clinical situation (…). The competent mental health professional acts in like fashion with regard to ongoing suicide risk” (p. 82).

Still, none of the included studies examined specific psychotherapeutic interventions or recommendations for the treatment of PSI. At present, for example, the recommendations that PSI should not be a reason for inpatient treatment (Paris 2007) or that it requires other interventions than acute suicidal ideation (Sansone 2004) are based on clinical experience rather than empirical knowledge. Furthermore, considerations that PSI can be addressed with the help of metacognitive therapy techniques (Teismann and Koban 2022) or techniques from Acceptance and Commitment Therapy (Hennings 2021) or dialectical behavior therapy (DBT; Paris 2007) are not supported by studies at this time, even though practitioners named them as approaches for treating PSI in clinical practice (Höller et al. 2025a). In this context, it should be noted that meta‐analytic studies have not been able to demonstrate any effect of DBT on the reduction of suicidal ideation (DeCou et al. 2019), which may also be related to the fact that the studies in question do not sufficiently differentiate between acute, recurrent, and persistent suicidal ideation. A pilot study showed that oral ketamine treatment is associated with a significant reduction in PSI (Can et al. 2021). However, PSI was insufficiently operationalized in this study, the follow‐up period was short, and the study sample was small. Far‐reaching generalizations are therefore not appropriate, and further research is needed on ketamine treatment for suicidal individuals (Feng et al. 2025; Wang et al. 2024). Interestingly, Dobscha et al. (2023) reported that mental health staff described that PSI conflicted with the treatment focus, as safety planning was frequently carried out during sessions. The primary care staff felt unprepared to evaluate risk in patients with PSI and the existing screening/assessment processes require modification for patients with PSI. Disentangling acute and persistent suicidal ideation appears to be highly challenging for clinicians (Sansone 2004) and can increase clinicians' insecurities when treating patients experiencing PSI (Höller et al. 2025b).

### 4.1 Limitations

A central limitation of this review lies in the inconsistent use of terminology across the literature. This variability may have resulted in relevant studies being overlooked during the selection process and carries the risk that researchers have a different understanding of the phenomenon. Furthermore, the deliberate decision to exclude case studies and theoretical contributions as well as the reliance on articles published in English constrained the scope of the review. Our decision to exclude studies that used a time criterion of < 3 months was based on the common time criteria for chronic conditions in the DSM‐5. It has to be noted that this initial selection of a time criterion was not based on evidence and is thus a matter of discretion (O'Halloran et al. 2004). Most importantly, the narrative nature of this review does not allow for the calculation of meta‐analytically derived effect sizes or prevalences.

### 4.2 Conclusions

Overall, the review demonstrates that a number of empirical studies have already addressed the phenomenon of PSI, predominantly employing longitudinal designs. Nevertheless, the lack of a consistent conceptualization and the heterogeneous operationalizations used across studies have produced highly diverse findings that are difficult to synthesize. To advance the field, future research requires greater conceptual clarity, beginning with a common definition and operational criteria (e.g., suicidal ideation being present for at least 12 months, occurring on most days; Teismann et al. 2025). Adequate methodological approaches such as ecological momentary assessments or daily diary designs are needed to provide fine‐grained insight into the epidemiological and phenomenological characteristics of PSI. Moreover, future work should systematically examine its associations with varying risk as well as protective factors. For the development of tailored treatment approaches (e.g., Teismann and Koban 2022), it appears crucial to explore potential intrapersonal functions of PSI (e.g., affect‐regulation; Brüdern et al. 2022; Kuehn et al. 2022) as well as interpersonal functions (Sansone 2004; Ronningstam et al. 2024).

## Funding

Open Access Publication was supported by the Open Access Publishing Fund of Leipzig University (project DEAL).

## Ethics Statement

The authors have nothing to report.

## Conflicts of Interest

The authors declare no conflicts of interest.

## Supporting information


**Table S1:** sltb70070‐sup‐0001‐TableS1.pdf.

## Data Availability

The authors have nothing to report.
