# Supplementary material for: Thinking About Suicide for a Long Time: A Scoping Review of Empirical Studies on Persistent Suicidal Ideation
Source: Suicide Life Threat Behav. 2025 Dec 16;55(6):e70070. doi: 10.1111/sltb.70070 (PMC12707180; doi:10.1111/sltb.70070)
Supplement: Supplementary file 1 — Table S1: sltb70070‐sup‐0001‐TableS1.pdf. [file SLTB-55-0-s001.pdf]

Supplementary table S1: Assessment of Suicidal Ideation in the included articles

Article(s)	Interview	Self-report questionnaire	Single items	Reference time
Willkens et al.; Dobscha et al		**PHQ-9** (Patient Health Questionnaire), item 9		past 14 days
Clark et al.		**SIDAS-M** (Suicidal Ideation Attributes Scale - Modified), item 1		past month
Kasckow, et al. (BSSI > = 0)<br>Seo, et al. (BSSI >=6)<br>Kivelä et al. (BSSI > 0)		**BSSI** (Beck Scale for Suicidal Ideation)		not reported
Guo et al.		**PANSI** (Positive and negative suicide ideation inventory)		not reported
Cohen et al.		**MCMI-II** (Millon Clinical Multiaxial Inventory II), item 79;59		Item 79: ... for many years...<br><br>Item 59: ... recently...
Köhler-Forsberg al.		**CHRT** (Concise Health Risk Tracking), item 10		not reported
Garakani et al.		**CSI-5** (Chronic Suicidal Ideation Scale), 5 modified Y-BOCS questions		Daily (none to >8 h/day or nearly constant)
Alonso et al.		**HDRS** (Hamilton Depression Rating Scale), item 3		not reported
Nobile et al.		**QIDS-SR** (Quick Inventory of Depressive Symptomatology). item 10 (of the **MARDS-SI**)		not reported
Nock et al.		**CSSRS** (Columbia Suicide Severity Rating Scale), "modified version"		lifetime ("for how many years...")
Gambadauro et al.		**PSS** (Paykel Suicide Scale)		past two weeks
Madsen et al.	**EPSIS - II** (European Parasuicide Study Interview Schedule), "individual items"			past year
Kim et al.		**K-YSR** (Korean Youth Self-Report), item 18; 91		previous 6 months
Buddeberg et al.		**MMPI** (Minnesota Multiphasic Personality Inventory), 7 items		T1 und T2: last 12 months<br><br>T3: last 6 months

Sasaki et al.			One single item: “Have you ever wanted to die from April 2020 to the present?”	Several months
Sicotte et al			Categorical clinical interview + chart review	past year
Vasiliadis et al			5 interview items:<br>„…period of at least two weeks when you thought you would be better off dead?“<br>„…period of 6 months when you thought you would be better off dead?“<br>„…did you seriously think to commit suicide or to take your own life?“<br>„Did you imagine a specific plan to commit suicide?“<br>„Did you attempt suicide or take your own life?“	past 12 months (suicide attempt, plan)<br>two weeks with SI<br>six months with SI
Knight et al	5 open questions (not explicitly mentioned)			not reported
Choi et al.			one single item: “Have you ever seriously thoughts about dying by suicide in the past year?“	12 months
Wolff et al.		**SIQ-JR** (Suicide Ideation Questionnaire – Junior)		past 30 days
Zhang et al.			2 items:<br>“… considered suicide?	past 12 months and lifetime
Gohar et al.	Clinical interview, single item: “: suicidal ideation … suicidal plans … or suicidal attempts …”			currently and previous 4 weeks
